# Supplementary material for: Birefringent Fourier filtering for single molecule coordinate and height super-resolution imaging with dithering and orientation
Source: Nat Commun. 2020 Oct 20;11:5307. doi: 10.1038/s41467-020-19064-6 (PMC7576605; doi:10.1038/s41467-020-19064-6)
Supplement: Supplementary file 1 — Supplementary Information [file 41467_2020_19064_MOESM1_ESM.pdf]

Birefringent Fourier filtering for single molecule Coordinate and Height super-resolution Imaging with Dithering and Orientation: Supplementary Information

Valentina Curcio[1], Luis A. Alemán Castañeda[1,2,*], Thomas G. Brown[2], Sophie Brasselet[1,*] & Miguel A. Alonso[1,2,*]

[1] *Aix Marseille Univ, CNRS, Centrale Marseille, Institut Fresnel, F-13013 Marseille, France*

[2] *The Institute of Optics, University of Rochester Rochester, NY 14627, U.S.A.*

**Corresponding authors: sophie.brasselet@fresnel.fr, miguel.alonso@fresnel.fr*

Content :

Supplementary Note 1: Theoretical description of the system

Supplementary Note 2: Orientation and Wobbling information

Supplementary Note 3: Theoretical study of precision and accuracy

Supplementary Note 4: Retrieval of parameters

Supplementary References

Supplementary Note 1: Theoretical description of the system

The goal of CHIDO is to image electric dipoles (fluorescing molecules) with a high NA microscope in order to determine their 3D location, orientation and wobbling from the location and shape of their PSFs. In this Supplementary Note we describe the forward model used to calculate these PSF shapes. Like for other methods, this model is based on the second moments of the dipole direction

from which the orientation-dependent variation of both the far-field[1] and the focused light at the detector[2–5] can be predicted.

Let the field at the system's pupil plane be denoted by $\mathbf{E}_{\text{pupil}}(\mathbf{u})$, where $\mathbf{u}$ is a dimensionless normalized pupil position with polar coordinates (u, φ) so that $u = 1$ corresponds to the edge of the pupil. For any orientation of a dipole, $\mathbf{E}_{\text{pupil}}$ can be expressed as a linear superposition of three fields, corresponding to the responses to electric dipoles oriented in the x, y (in-plane) and z (out-of-plane) directions. The field is highly collimated at the pupil plane, so its z component is unimportant. If the dipole is shifted in z from the nominal object plane, the field at the pupil acquires a chirp factor whose phase is proportional to this displacement, while displacements in x and y introduce a linear phase factor. Let us for now ignore these transverse displacements and focus on the axial displacement and the orientation of the dipole. The field at the pupil can then be written as

$$\mathbf{E}_{\text{pupil}}(\mathbf{u}) = \sum_i E_i \mathbf{K}_i(\mathbf{u}) \exp[-\mathrm{i}k\mathrm{n}z\gamma(u)], \tag{1}$$

where E_i is the ith component ($i = x, y, z$) of the field generated by the molecule, $\mathbf{K}_i(\mathbf{u})$ is the amplitude and polarization distribution at the pupil plane generated by each of these components and assumed to differ from zero only for $u \leq 1$, k is the wavenumber, n is the refractive index of the medium embedding the fluorophores, and for an aplanatic system $\gamma(u) = (1 - u^2 \sin^2 \theta_0)^{1/2}$ with θ_0 being the entrance half-angle of the objective lens.

If the imaging system is axially symmetric, the functions $\mathbf{K}_i(\mathbf{u})$ must take the general form

$$\mathbf{K}_x(\mathbf{u}) = \hat{\mathbf{x}}\, g_0(u) + (\hat{\mathbf{x}}\cos 2\varphi + \hat{\mathbf{y}}\sin 2\varphi) g_2(u), \tag{2a}$$

$$\mathbf{K}_y(\mathbf{u}) = \hat{\mathbf{y}}\, g_0(u) + (\hat{\mathbf{x}}\sin 2\varphi - \hat{\mathbf{y}}\cos 2\varphi) g_2(u), \tag{2b}$$

$$\mathbf{K}_z(\mathbf{u}) = (\hat{\mathbf{x}}\cos\varphi + \hat{\mathbf{y}}\sin\varphi) g_1(u), \tag{2c}$$

where, if the system is also aplanatic, the functions $g_n(u)$ are given (to within an unimportant constant factor) by

$$g_{0,2}(u) = \frac{t_{\mathrm{p}}(u)\gamma(u) \pm t_{\mathrm{s}}(u)}{2\sqrt{\gamma(u)}}, \tag{3a}$$

$$g_1(u) = \frac{\sin\theta_0\, u\, t_{\mathrm{p}}(u)}{\sqrt{\gamma(u)}}, \tag{3b}$$

with $t_{\mathrm{p}}(u)$ and $t_{\mathrm{s}}(u)$ being the transmission coefficients for the radial and azimuthal components, respectively. These coefficients account not only for interfaces inside the microscope, but also for passage from the medium containing the fluorophores to glass to the immersion oil being used.

The basis of this method is to use a stress-engineered optical element (SEO) for wavefront coding at the pupil. This element is described by the Jones matrix $\mathbb{J}(\mathbf{u})$ given in Eq. (1) of the main text. After passing through the SEO, the field at the pupil is given by

$$\mathbf{E}_{\mathrm{SEO}}(\mathbf{u}) = \mathbb{J}(\mathbf{u})\mathbf{E}_{\mathrm{pupil}}(\mathbf{u}). \tag{4}$$

The field is then focused to form an image. This focusing corresponds to Fourier transformation:

$$\mathbf{E}_{\mathrm{det}}(\boldsymbol{\rho}) = \frac{R^2}{\lambda f}\int \mathbf{E}_{\mathrm{SEO}}(\mathbf{u}) \exp\left(-\mathrm{i}\frac{kR\,\mathbf{u}\cdot\boldsymbol{\rho}}{f}\right) \mathrm{d}^2 u = \mathcal{F}\mathbf{E}_{\mathrm{SEO}}, \tag{5}$$

where R is the physical radius of the pupil, f is the focal length of the focusing system, and $\boldsymbol{\rho} = (\rho\cos\phi, \rho\sin\phi)$ are the coordinates at the detector plane. In what follows we use the shorthand

$\mathcal{F}$ for the Fourier transformation from the pupil to the detector plane that includes these physical parameters.

As mentioned in the main body, CHIDO relies on forming separate images for the two circular polarization components emerging from the SEO, by using a combination of a quarter-wave plate and a Wollaston prism. In principle, each of these images would be sufficient to determine the orientation of the emitter if its z coordinate were known. However, using both images allows decoupling the effects of orientation and z displacements on the PSFs, which is a main feature of this technique. It also allows utilizing all photons, leading to more accurate estimates, which is particularly important when photons are scarce.

To make the following analysis general, we consider separation into any two orthogonal polarization components, represented by the unit vectors $\hat{\mathbf{p}}_p$ for $p = 1, 2$. The corresponding intensity images at the two regions of the CCD are then given by

$$I^{(p)}(\boldsymbol{\rho}) = \sum_{i,j} \Gamma_{ij} G_i^{(p)*}(\boldsymbol{\rho}) G_j^{(p)}(\boldsymbol{\rho}), \tag{6}$$

where $\Gamma_{ij} = \langle E_i^* E_j \rangle$ (in the x, y, z basis) is an element of the correlation matrix $\boldsymbol{\Gamma}$ of the different components of the (possibly wobbling) dipole source, and

$$G_i^{(p)}(\boldsymbol{\rho}) = \mathcal{F}\{\hat{\mathbf{p}}_p^* \cdot \mathbb{J}(\mathbf{u}) \cdot \mathbf{K}_i(\mathbf{u}) \exp[-\mathrm{i}kz\gamma(u)]\}. \tag{7}$$

As described in Eq. (3) of the main text, the intensity corresponding to each polarization can be expressed as a sum of a basis of PSFs $\mathcal{I}_n^{(p)}$ weighted by the generalized Stokes parameters S_n

of the 3×3 correlation of the emitter. The expressions for these PSF basis elements are given by

$$\mathcal{I}_0^{(p)}(\boldsymbol{\rho}) = \frac{\left|G_x^{(p)}(\boldsymbol{\rho})\right|^2 + \left|G_y^{(p)}(\boldsymbol{\rho})\right|^2 + \left|G_z^{(p)}(\boldsymbol{\rho})\right|^2}{\sqrt{3}}, \tag{8a}$$

$$\mathcal{I}_1^{(p)}(\boldsymbol{\rho}) = \left|G_x^{(p)}(\boldsymbol{\rho})\right|^2 - \left|G_y^{(p)}(\boldsymbol{\rho})\right|^2, \tag{8b}$$

$$\mathcal{I}_2^{(p)}(\boldsymbol{\rho}) = 2\,\mathrm{Re}\left[G_x^{(p)*}(\boldsymbol{\rho})G_y^{(p)}(\boldsymbol{\rho})\right], \tag{8c}$$

$$\mathcal{I}_3^{(p)}(\boldsymbol{\rho}) = 2\,\mathrm{Im}\left[G_x^{(p)*}(\boldsymbol{\rho})G_y^{(p)}(\boldsymbol{\rho})\right], \tag{8d}$$

$$\mathcal{I}_4^{(p)}(\boldsymbol{\rho}) = 2\,\mathrm{Re}\left[G_x^{(p)*}(\boldsymbol{\rho})G_z^{(p)}(\boldsymbol{\rho})\right], \tag{8e}$$

$$\mathcal{I}_5^{(p)}(\boldsymbol{\rho}) = 2\,\mathrm{Im}\left[G_x^{(p)*}(\boldsymbol{\rho})G_z^{(p)}(\boldsymbol{\rho})\right], \tag{8f}$$

$$\mathcal{I}_6^{(p)}(\boldsymbol{\rho}) = 2\,\mathrm{Re}\left[G_y^{(p)*}(\boldsymbol{\rho})G_z^{(p)}(\boldsymbol{\rho})\right], \tag{8g}$$

$$\mathcal{I}_7^{(p)}(\boldsymbol{\rho}) = 2\,\mathrm{Im}\left[G_y^{(p)*}(\boldsymbol{\rho})G_z^{(p)}(\boldsymbol{\rho})\right], \tag{8h}$$

$$\mathcal{I}_8^{(p)}(\boldsymbol{\rho}) = \frac{\left|G_x^{(p)}(\boldsymbol{\rho})\right|^2 + \left|G_y^{(p)}(\boldsymbol{\rho})\right|^2 - 2\left|G_z^{(p)}(\boldsymbol{\rho})\right|^2}{\sqrt{3}}. \tag{8i}$$

Suppplementary Fig. 1 shows these elementary PSFs and their derivatives in z for $z = 0$, where unlike in the main text we show also the ones associated with helicity ($n = 3, 5, 7$), which are not used for dipole orientation.

Let us now discuss the chromatic dependence of the PSFs. Note that the treatment above assumes monochromatic illumination. However, the fluorescent emission from the molecules is not strictly monochromatic but includes a spectral range of about 40 nm around a peak wavelength of 519 nm after passing through a fluorescence filter. The calculation of the PSF components $\mathcal{I}_n^{(p)}$ then requires the superposition of the corresponding PSFs weighted by the spectrum. It turns out, nevertheless, that the parameter c is approximately inversely proportional to the wavelength because

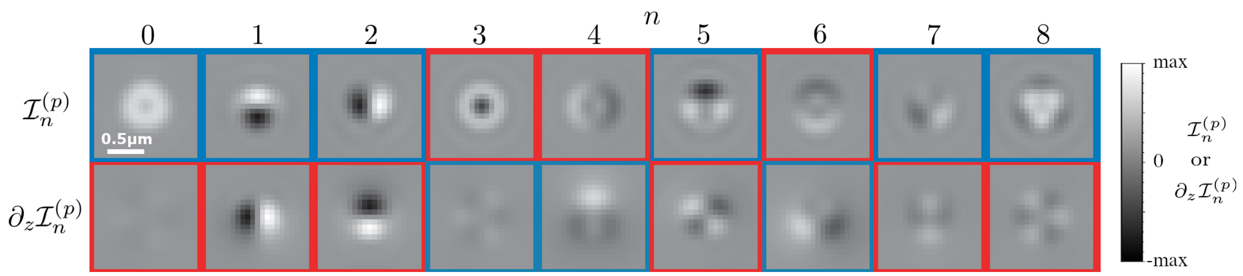

Supplementary Figure 1: Complete set of theoretical PSF components in CHIDO imaging. The figure shows both $\mathcal{I}_n^{(p)}$ and $\partial_z \mathcal{I}_n^{(p)}$ for $c = \pi$, $z = 0$, and $p = \mathcal{R}$. The corresponding components for $p = \mathcal{L}$ are identical, except that those surrounded by red boxes would have the opposite sign. Each row is normalized separately as their units are different.

it characterizes a phase retardance. This spectral dependence then balances out that resulting from the presence of the wavenumber in the exponent of the kernel of the Fourier transform in Eq. (5) for propagating from the pupil to the image plane, making the contributions from each spectral component largely consistent. The resulting PSF components, over a 100 nm spectral expansion, are seen to be very similar to those corresponding to the peak wavelength. This is illustrated in Supplementary Fig. 2 for the six relevant PSF components at $z = 0$. We can see that even if the fluorescence filter is not used, the chromatically-integrated PSF elements are very similar to those corresponding to the nominal wavelength. Note that this property stays valid for an extent in z of about 500 nm, above which the errors on the PSF can surpass 5% because the rate of rotation of the PSFs with defocus depends approximately on the ratio z/λ. A reduction of the detected spectral width down to 40 nm (which is the case in the present experiments) permits to benefit from the achromaticity property of CHIDO even at large shifts in z.

As explained in the main text, it is assumed that the fluorescent molecules have no chiral-

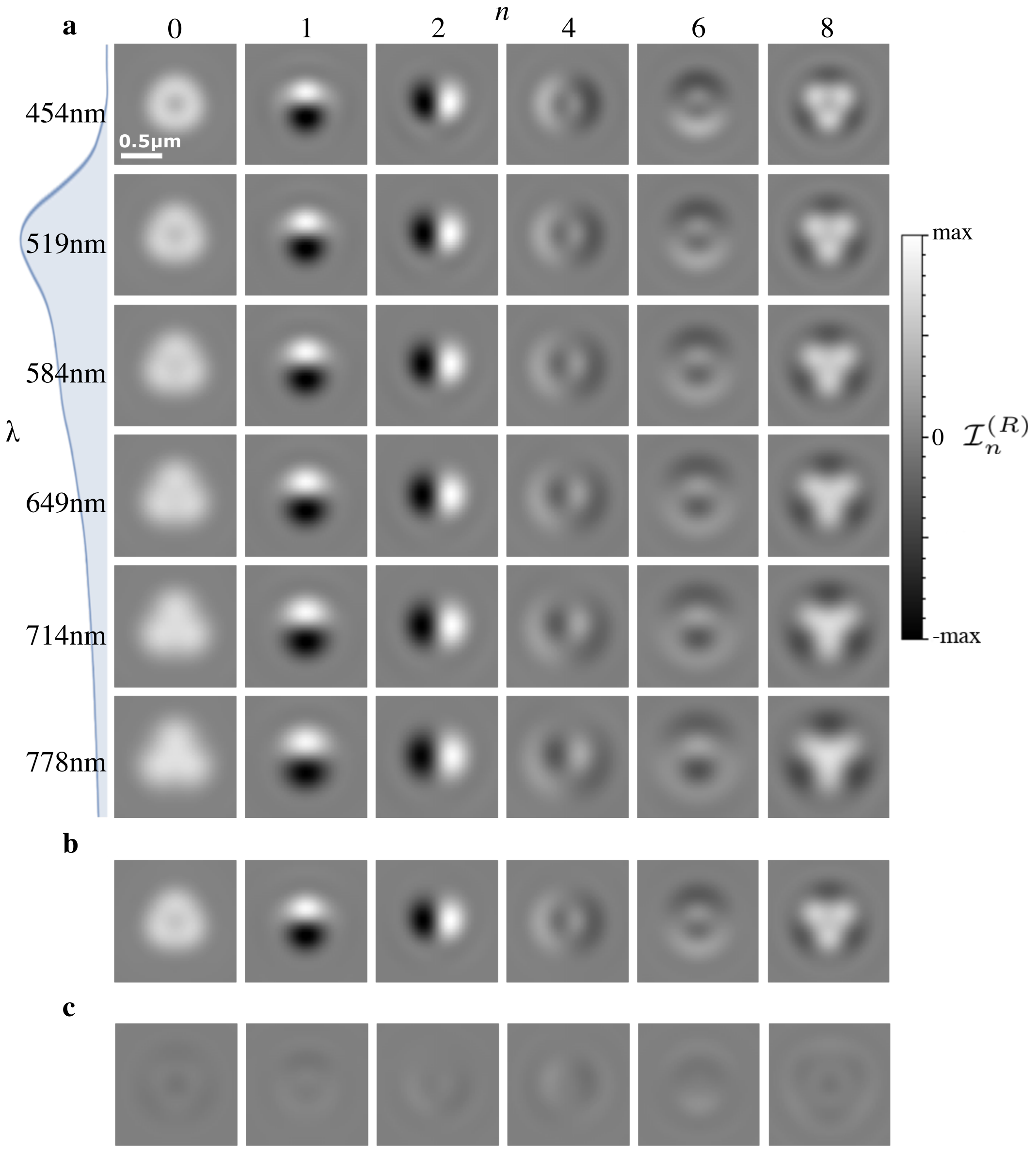

Supplementary Figure 2: Chromatic dependence of the PSF components. (a) Theoretically calculated PSF components $\mathcal{I}_n^{(\mathcal{R})}$ at $z = 0$ for a broad range of wavelengths, where $c = \pi$ at the nominal wavelength of 519nm. Each row is normalized separately. The fluorescence spectrum is shown on the left. (b) Superposition of these PSF components weighted by the whole fluorescence spectrum. (c) Difference between the polychromatic PSFs in (b) and those for the nominal (peak) wavelength shown in the second row of (a).

ity, so that $\mathcal{I}_3^{(p)}$, $\mathcal{I}_5^{(p)}$ and $\mathcal{I}_7^{(p)}$ are not required in the retrieval of the localization, orientation and wobbling of the molecules. Note, however, that as mentioned in the main text it is convenient for the alignment of the system to use measurements of fluorescent beads where a circular polarizer is inserted before the SEO. In this case, the PSFs are dominated by $\mathcal{I}_0^{(p)}$ and $\mathcal{I}_3^{(p)}$, which are approximately rotationally symmetric and whose combination produces PSFs that are a donut shape and a spot. These shapes vary slowly with defocus around the nominal plane. The alignment of the system and the calibration of residual birefringence prior to the SEO are then adjusted to maximize the rotational symmetry of the measured PSFs.

We now study the dependence of the z coordinate of the emitter on the PSFs and the information they carry. First, notice that this z dependence justifies the choice of separating the image in terms of circular polarization components. For example, if no QWP were used, the Wollaston prism would separate two orthogonal linear polarizations. In this case, however, the PSF components $\mathcal{I}_3^{(p)}$, $\mathcal{I}_4^{(p)}$ and $\mathcal{I}_6^{(p)}$ would vanish for $z = 0$, and it would not be possible to determine the corresponding generalized Stokes parameters. On the other hand, decomposing the images in terms of circular polarizations leads to PSFs that depend strongly on all the relevant parameters, as

we now see. For right ($\mathcal{R}$) and left ($\mathcal{L}$) circular polarizations, the functions in Eq. (7) become

$$
\begin{aligned}
G_x^{(\mathcal{R}/\mathcal{L})}(\boldsymbol{\rho}) &= \frac{1}{\sqrt{2}}\mathcal{F}(\exp(-\mathrm{i}kz\gamma)\{\mathcal{C}[g_0 - g_2\exp(\mp 2\mathrm{i}\varphi)] \\
&\quad + \mathrm{i}\mathcal{S}\exp(\pm\mathrm{i}\varphi)[g_0 - g_2\exp(\pm 2\mathrm{i}\varphi)]\}), \qquad (9a) \\
G_y^{(\mathcal{R}/\mathcal{L})}(\boldsymbol{\rho}) &= \mp\frac{1}{\sqrt{2}}\mathcal{F}(\exp(-\mathrm{i}kz\gamma)\{\mathrm{i}\mathcal{C}[g_0 + g_2\exp(\mp 2\mathrm{i}\varphi)] \\
&\quad + \mathcal{S}\exp(\pm\mathrm{i}\varphi)[g_0 + g_2\exp(\pm 2\mathrm{i}\varphi)]\}), \qquad (9b) \\
G_z^{(\mathcal{R}/\mathcal{L})}(\boldsymbol{\rho}) &= \frac{1}{\sqrt{2}}\mathcal{F}\{\exp(-\mathrm{i}kz\gamma)g_1 \\
&\quad \times [\mathcal{C}\exp(\mp\mathrm{i}\varphi) + \mathrm{i}\mathcal{S}\exp(\pm 2\mathrm{i}\varphi)]\} \qquad (9c)
\end{aligned}
$$

where $\mathcal{C} = \cos(cu/2)$ and $\mathcal{S} = \sin(cu/2)$. By inserting these expressions into Eqs. (8), one can find the PSF components for any axial displacement z.

While the best retrieval results might be achieved by using the rigorous dependence in z of the model, approximations can be made to find a simplified parametrization in z of the form

$$
\mathcal{I}_n^{(p)}(\boldsymbol{\rho}, z) \approx \sum_{m=0}^{M} h_{n,m}(z)\,\mathcal{I}_{n,m}^{(p)}(\boldsymbol{\rho}). \qquad (10)
$$

where the components $\mathcal{I}_{n,m}^{(p)}$ and the functions $h_{n,m}(z)$ are found by fitting over calculations at different z or from calibration experimental data. For example, for a given n, a singular value decomposition can be used to fit $\mathcal{I}_n^{(p)}$ sampled over several values of z and the leading M terms in this decomposition can be used to approximate the expression. The resulting eigenvectors over the samples in z can be used to find fits for the functions $h_{n,m}(z)$. For small displacements ($|z|\stackrel{<}{\sim}\lambda/\mathrm{n}\cos\theta_0$) it can be sufficient to use monomials $h_{n,m}(z) = z^m$, the constant and the linear functions being enough to capture the main features of the transformation. We used this approach in the proof-of-principle experiments with fluorescent beads simulating molecules with different orientations,

where the PSF model was constructed from experimental measurements. A quadratic term was included in order to help avoid the resulting approximate PSFs from containing negative values.

Supplementary Note 2: Orientation and wobbling information

As mentioned earlier, the generalized Stokes parameters can be measured experimentally by finding the coefficients of the PSF basis in order to match as well as possible the measured PSF at the two detector regions. From these parameters, we can extract information about the dipole's orientation and wobbling, both in the xy-plane and out of it. Consider first the case of a dipole whose orientation wobbles around the z axis with main directions aligned with the x and y axes, so that its direction cosines in the x and y directions have standard deviations Δ_1 and Δ_2, respectively. These standard deviations characterize the half-angles of the elliptical cone of directions within which the molecule wobbles. The components of the correlation matrix are then

$$\mathbf{\Gamma}_0 = |E_0|^2 \begin{pmatrix} \Delta_1^2 & 0 & 0 \\ 0 & \Delta_2^2 & 0 \\ 0 & 0 & 1-\Delta_1^2-\Delta_2^2 \end{pmatrix}. \tag{11}$$

For a molecule with arbitrary orientation, the generic correlation matrix corresponds to a 3D rotation of this matrix:

$$\mathbf{\Gamma} = \mathbf{R}\mathbf{\Gamma}_0\mathbf{R}^{\mathrm{T}}, \tag{12}$$

where $\mathbf{R}$ is a 3×3 rotation matrix. Since both $\mathbf{\Gamma}_0$ and $\mathbf{R}$ are real, so is $\mathbf{\Gamma}$, which means that $S_3 = S_5 = S_7 = 0$. That is, as mentioned earlier, only six generalized Stokes parameters are relevant to this problem, of which one, $S_0 = |E_0|^2/\sqrt{3}$, is independent of orientation and hence

serves only as normalization for the remaining five nonzero parameters. The parameters S_3, S_5, and S_7 could be useful, e.g., in the measurement of chiral molecules for which $\mathbf{\Gamma}$ can be complex, but this is not the case of the molecules studied here.

The retrieval of the dipole's main orientation angles θ and ξ, of the standard deviations Δ_1 and Δ_2, and their corresponding directions of vibration can be achieved by simply finding the eigenvalues and eigenvectors of the estimation of $\mathbf{\Gamma}$ resulting from the measurements. Let these eigenvalues and eigenvectors be denoted by Λ_i and $\mathbf{v}_i$, respectively, for $i = 1, 2, 3$, and ordered such that $\Lambda_1 \geq \Lambda_2 \geq \Lambda_3$. Ideally, the molecule's average orientation is given by $\mathbf{v}_1$, and $\Delta_1^2 = \Lambda_2/T$, $\Delta_2^2 = \Lambda_3/T$, where $T = \mathrm{Tr}(\mathbf{\Gamma}) = \Lambda_1 + \Lambda_2 + \Lambda_3$. The directions of oscillation associated with these two variances are those of $\mathbf{v}_2$ and $\mathbf{v}_3$, respectively, so it is possible in theory to estimate the asymmetry of the wobbling. In practice, however, limitations due to small numbers of photons, additive noise and pixelation introduce errors in these measures. The effect of these sources of error on the eigenvectors and eigenvalues $\mathbf{v}_i, \Lambda_i$ is larger for larger i (i.e., for smaller Λ_i), which means that the estimation of the main orientation $\mathbf{v}_i$ is significantly more robust than that of the wobbling, particularly for small wobbling angles. Ideally, the polarization matrix should be non-negative definite, i.e., $\Lambda_i \geq 0$. However, the sources of error mentioned earlier can make the estimated Λ_3 and sometimes even Λ_2 negative. To alleviate this problem, we make the assumption that the wobbling of the dipole is isotropic with respect to the average dipole direction (that is $\Delta_1 = \Delta_2 = \Delta$) so we use the largest (and more numerically robust) of the two estimated eigenvalues, namely $\Delta^2 = \Lambda_2/T$. In this case, the directions $\mathbf{v}_{2,3}$ are no longer necessary, and the only meaningful directional parameters are the polar and azimuthal angles, θ and ξ, characterizing the direction of

the dipole, as well as the amount of wobbling, Δ. For ease of interpretation, this last quantity can be transformed into a cone angle δ or a solid angle Ω, corresponding to the assumption that the dipole wobbles within this cone with equal probability of being in any direction. A straightforward calculation gives

$$\frac{\Lambda_2}{T} = \frac{3}{8} - \frac{[\cos(\delta/2) + 1/2]^2}{6} = \frac{3}{8} - \frac{[3/2 - \Omega/2\pi]^2}{6}, \tag{13}$$

from where we can find

$$\Omega = 3\pi \left(1 - \sqrt{1 - \frac{8}{3}\frac{\Lambda_2}{T}}\right), \tag{14}$$

so that $\Lambda_2 = 0$ corresponds to no wobbling ($\delta, \Omega = 0$) while the opposite extreme of the largest possible value $\Lambda_2 = T/3$ (assuming $\Lambda_3 = \Lambda_2$) gives isotropic wobbling in all directions corresponding to $\delta = 180^\circ$ and $\Omega = 2\pi$. Note that in theory the largest value Λ_2 can take is $T/2$, which violates the assumption of isotropic wobbling but is numerically possible even for isotropic wobbling due to noise or to errors in the reference PSF basis. To prevent the unphysical results for Ω that Eq. (14) would give, we constrain the values of Λ_2/T to the interval $[0, 1/3]$, so that when the retrieved value is outside this interval we simply use the closest value within it. Also, we found through simulations that the results can be improved if an extra step is added, consisting of using the retrieved values as starting points for maximizing the correlation of the measured PSFs to the model constrained to isotropic wobbling. The approach of maximization of the correlation was used for retrieving the parameters in the STORM measurements.

The so-called rotational mobility or rotational constraint, which describes the amount of wobble of a single fluorophore, has been studied both in its 2D[6,7] and 3D[4,7,8] forms. Following the notation in those references, we denote this parameter here as $\gamma_{2D/3D}$, depending on the dimen-

sionality. The upper bound for this parameter, $\gamma_{\mathrm{D}} = 1$, implies that the molecule is completely fixed, or at least that the wobbling is in a time scale much larger than the integration time of the detector. On the contrary, the lower bound $\gamma_{\mathrm{D}} = 0$ means that the molecule wobbles freely and isotropically within the integration time of the detector. The relation between the measures of polarization $P_{\mathrm{2D/3D}}$ and the mobility parameter, $\gamma_{\mathrm{2D/3D}}$, is now described. As mentioned earlier, the second-moment (or polarization) matrix $\mathbf{\Gamma}$ (denoted as $\mathbf{M}$ by other authors) can be written as

$$\mathbf{\Gamma} = \sum_{j=1}^{3} \Lambda_j \mathbf{v}_j \mathbf{v}_j^{\dagger}. \tag{15}$$

Let us first consider the 2D case in which the molecule is taken to wobble only within the xy-plane. We can then use the the submatrix

$$\mathbf{\Gamma}_{xy} = \sum_{j=1}^{2} \tilde{\Lambda}_j \tilde{\mathbf{v}}_j \tilde{\mathbf{v}}_j^{\dagger}, \tag{16}$$

where $\tilde{\Lambda}_j$ are the eigenvalues of the sub-matrix, $\tilde{\mathbf{v}}_j$ their corresponding eigenvectors, and we assume $\tilde{\Lambda}_1 \geq \tilde{\Lambda}_2$. Notice that in general $\tilde{\Lambda}_j \neq \Lambda_j$. We can express our submatrix as[6,7]

$$\mathbf{\Gamma}_{xy} = (\tilde{\Lambda}_1 + \tilde{\Lambda}_2) \left\{ \gamma_{\mathrm{2D}} (\mathbf{v}_1 \mathbf{v}_1^{\dagger}) + \frac{(1 - \gamma_{\mathrm{2D}})}{2} \mathbf{I} \right\}, \tag{17}$$

where the rotational mobility parameter in 2D is defined as

$$\gamma_{\mathrm{2D}} = \frac{\tilde{\Lambda}_1 - \tilde{\Lambda}_2}{\tilde{\Lambda}_1 + \tilde{\Lambda}_2} \tag{18}$$

which corresponds exactly to the definition of degree of polarization for paraxial fields[9], P_{2D}. Further, if the dipole wobbles uniformly within an angle[6] δ_{2D}, the mobility parameter ends up being

$$\gamma_{\mathrm{2D}} = \frac{\sin \delta_{\mathrm{2D}}}{\delta_{\mathrm{2D}}}. \tag{19}$$

The situation changes when considering the 3D problem. For this scenario, a decomposition for $\mathbf{\Gamma}$ analogous to that in Eq. (17) was proposed[7]:

$$\mathbf{\Gamma} = (\Lambda_1 + \Lambda_2 + \Lambda_3)\left\{\gamma_{3\mathrm{D}}(\mathbf{v}_1\mathbf{v}_1^\dagger) + \frac{(1-\gamma_{3\mathrm{D}})}{3}\mathbf{I} + \frac{\Lambda_2 - \Lambda_3}{2(\Lambda_1 + \Lambda_2 + \Lambda_3)}(\mathbf{v}_2\mathbf{v}_2^\dagger - \mathbf{v}_3\mathbf{v}_3^\dagger)\right\}, \tag{20}$$

where the 3D rotational mobility parameter is given by

$$\gamma_{3\mathrm{D}} = \frac{2\Lambda_1 - \Lambda_2 - \Lambda_3}{2(\Lambda_1 + \Lambda_2 + \Lambda_3)}. \tag{21}$$

The first term in Eq. (20) corresponds to the contribution of a completely fixed dipole (or main direction of orientation if there is wobble). The second term, which is proportional to the identity matrix, corresponds to the amount of isotropic wobbling. The third term characterizes the possible anisotropy in the rotational mobility, meaning that the dipole would wobble inside a cone with elliptical profile rather than circular.

Notice that this rotational mobility does not correspond in general to the degree of polarization[10–13] used in the main manuscript, which is defined as

$$P_{3\mathrm{D}} = \left[\frac{3\,\mathrm{tr}\mathbf{\Gamma}^2}{2\,(\mathrm{tr}\mathbf{\Gamma})^2}. - \frac{1}{2}\right]^{1/2} = \frac{(\Lambda_1^2 + \Lambda_2^2 + \Lambda_3^2 - \Lambda_1\Lambda_2 - \Lambda_1\Lambda_3 - \Lambda_2\Lambda_3)^{1/2}}{\Lambda_1 + \Lambda_2 + \Lambda_3}. \tag{22}$$

Nevertheless, if we assume that the dipole wobbles isotropically (a common assumption in the literature[4,6,7]) within a circular cone subtending an angle $\delta_{3\mathrm{D}}$, or solid angle $\Omega_{3\mathrm{D}}$, the two lowest eigenvalues coincide and Eqs. (21) and (22) agree:

$$\Lambda_2 = \Lambda_3 \implies \gamma_{3\mathrm{D}} = P_{3\mathrm{D}}.$$

By using Eqs. (22) and (21) we can find the general relation between these two parameters:

$$P_{3\mathrm{D}} = \gamma_{3\mathrm{D}}\sqrt{1 + 3\left(\frac{\Lambda_2 - \Lambda_3}{2\Lambda_1 - \Lambda_2 - \Lambda_3}\right)^2}, \tag{23}$$

which indicates that $P_{3\mathrm{D}} \geq \gamma_{3\mathrm{D}}$, the equality holding only for wobbling that is isotropic (in the second-order sense) around a main direction.

Supplementary Note 3: Theoretical study of precision and accuracy

We now study theoretically the precision and accuracy of CHIDO. To do this, we first study the Cramér-Rao (CR) lower bounds for the estimated parameters. Let us start by considering only the directional parameters. Let $\mathcal{I}$ denote the intensity distribution over the two detectors, which is given by

$$\mathcal{I} = \sum_{n=0}^{8} S_n \mathcal{I}_n, \tag{24}$$

where, as mentioned earlier, $S_3 = S_5 = S_7 = 0$. The sum over all pixels on the two channels gives

$$\langle \mathcal{I} \rangle = \sum_{n=0}^{8} S_n \langle \mathcal{I}_n \rangle \approx S_0 \langle \mathcal{I}_0 \rangle, \tag{25}$$

where in this section angular brackets denote a sum over all pixels. In the last step we used the fact that $\langle \mathcal{I}_{1,2} \rangle = 0$ due to the anti-parity of these PSF elements over each channel ($\mathcal{R}$ and $\mathcal{L}$), $\langle \mathcal{I}_{4,6} \rangle = 0$ due to the change of sign for the corresponding PSF elements between the channels $\mathcal{R}$ and $\mathcal{L}$, and for appropriate choices of c the positive and negative contributions in $\mathcal{I}_8$ balance sufficiently well that $|\langle \mathcal{I}_8 \rangle|$ is sufficiently smaller than $\langle \mathcal{I}_0 \rangle$. For example, for $c = 1.2\pi$, $|\langle \mathcal{I}_8 \rangle|$ is about a fifth of $\langle \mathcal{I}_0 \rangle$, which while not orders of magnitude smaller, is sufficiently small to make the simple estimates that result from neglecting it meaningful.

Let us consider first the case in which there is no background intensity. The probability

density distribution $\mathcal{P}$ as a function of the normalized Stokes parameters $s_n = S_n/S_0$ is

$$\mathcal{P} \approx \frac{\mathcal{I}_0 + \sum_{n=1}^{8} s_n \mathcal{I}_n}{\langle \mathcal{I}_0 \rangle}, \tag{26}$$

Before finding the Fisher information with respect to the angular parameters ξ, θ, Ω, it is useful to estimate the Fisher information matrix with respect to the normalized generalized Stokes parameters s_n. Given the linear dependence of $\mathcal{I}$ on these parameters, we find that the elements of this matrix are

$$\mathcal{J}^{\text{Stokes}}_{s_n s_{n'}} = \mathcal{N} \left\langle \frac{\partial_{s_n} \mathcal{P}\, \partial_{s_{n'}} \mathcal{P}}{\mathcal{P}} \right\rangle \approx \frac{\mathcal{N}}{\langle \mathcal{I}_0 \rangle} \left\langle \frac{\mathcal{I}_n \mathcal{I}_{n'}}{\mathcal{I}_0 + \sum_{n=1}^{8} s_n \mathcal{I}_n} \right\rangle, \tag{27}$$

for $n, n' = 1, 2, ..., 8$, and where $\mathcal{N}$ is the number of photons in the measured PSF. It turns out that a good order-of-magnitude estimate can be obtained by ignoring the part in the denominator that depends on s_n, leading to

$$\mathcal{J}^{\text{Stokes}}_{s_n s_{n'}} \approx \frac{\mathcal{N}}{\langle \mathcal{I}_0 \rangle} \left\langle \frac{\mathcal{I}_n \mathcal{I}_{n'}}{\mathcal{I}_0} \right\rangle, \tag{28}$$

From this approximation, we can appreciate the usefulness of the PSF components $\mathcal{I}_n$ being approximately orthogonal (for appropriate choices of c): the Fisher information matrix is then approximately diagonal, namely

$$\mathcal{J}^{\text{Stokes}} \approx \mathcal{N} \text{Diag}(a_1, a_2, a_3, a_4, a_5, a_6, a_7, a_8). \tag{29}$$

For the values of c used here, the only nondiagonal elements of the Fisher information matrix that are not orders of magnitude smaller than the diagonal ones are $\mathcal{J}^{\text{Stokes}}_{15}$ and $\mathcal{J}^{\text{Stokes}}_{27}$, which are not relevant here since $s_5 = s_7 = 0$. For $c = 1.2\pi$, the relevant diagonal elements are approximately $a_1 \approx a_2 \approx 0.63$, $a_4 \approx a_6 \approx 0.41$, and $a_8 \approx 0.47$.

Note that s_n can be parametrized in terms of the directional parameters ξ, θ, Ω as

$$s_1 = \frac{\sqrt{3}}{2} P_{3D} \sin^2\theta \cos 2\xi, \tag{30a}$$

$$s_2 = \frac{\sqrt{3}}{2} P_{3D} \sin^2\theta \sin 2\xi, \tag{30b}$$

$$s_4 = \frac{\sqrt{3}}{2} P_{3D} \sin 2\theta \cos\xi, \tag{30c}$$

$$s_6 = \frac{\sqrt{3}}{2} P_{3D} \sin 2\theta \sin\xi, \tag{30d}$$

$$s_8 = -P_{3D} \frac{1 + 3\cos 2\theta}{4}. \tag{30e}$$

We can then calculate the Fisher information matrix for the directional variables as

$$\mathcal{J}^{\text{angular}}_{\alpha_i \alpha_{i'}} = \sum_{n,n'} \mathcal{J}^{\text{Stokes}}_{s_n s_{n'}} (\partial_{\alpha_i} s_n)\,(\partial_{\alpha_{i'}} s_{n'}), \tag{31}$$

where $\alpha_{1,2,3} = \xi, \theta, \Omega$. By using the diagonal approximation for $\mathcal{J}^{\text{Stokes}}$, we can find very simple approximate expressions for the angular standard deviations:

$$\sigma_\xi \approx \frac{(4\pi)^2}{(8\pi^2 - 6\pi\Omega + \Omega^2)\sin\theta\sqrt{6\mathcal{N}(u_+ - u_- \cos 2\theta)}}, \tag{32a}$$

$$\sigma_\theta \approx \frac{2(4\pi)^2}{(8\pi^2 - 6\pi\Omega + \Omega^2)\sqrt{6\mathcal{N}(v_+ - v_- \cos 4\theta)}}, \tag{32b}$$

$$\sigma_\Omega \approx \frac{2(4\pi)^2}{(3\pi - \Omega)\sqrt{2\mathcal{N}(w - 12u_- \cos 2\theta + 3v_- \cos 4\theta)}}, \tag{32c}$$

with $u_\pm = a_1 \pm a_4$, $v_\pm = a_1 \pm 4a_4 + 3a_8$, and $w = 9a_1 + 12a_4 + 11a_8$. Notice that these estimates are independent of ξ and, if all a_n were equal, the dependence in θ of these estimates would disappear except for the inverse dependence on $\sin\theta$ for σ_ξ. Even when these coefficients are not exactly equal, the terms involving u_-, v_- are considerably smaller and can be neglected, leading to even

simpler estimates:

$$\sigma_\xi \approx \frac{(4\pi)^2}{(8\pi^2 - 6\pi\Omega + \Omega^2)\sin\theta\sqrt{6\mathcal{N}}}, \tag{33a}$$

$$\sigma_\theta \approx \frac{(4\pi)^2}{(8\pi^2 - 6\pi\Omega + \Omega^2)\sqrt{6\mathcal{N}}}, \tag{33b}$$

$$\sigma_\Omega \approx \frac{(4\pi)^2}{2.8(3\pi - \Omega)\sqrt{\mathcal{N}}}. \tag{33c}$$

Note that, in arriving at these simple approximations, we rounded up numerical factors coming from u_+ and v_+, so that $\sigma_\xi \sin\theta \approx \sigma_\theta$. If the numerical quantities are calculated more precisely one finds that σ_θ is slightly larger than $\sigma_\xi \sin\theta$, and this is reflected in the rigorously computed CR lower bounds presented in the main manuscript. These expressions become even simpler when expressed in terms of P_{3D}:

$$\sigma_\xi \approx \frac{2}{P_{\mathrm{3D}}\sin\theta\sqrt{6\mathcal{N}}}, \tag{34a}$$

$$\sigma_\theta \approx \frac{2}{P_{\mathrm{3D}}\sqrt{6\mathcal{N}}}, \tag{34b}$$

$$\sigma_{P_{\mathrm{3D}}} \approx \frac{1.43}{\sqrt{\mathcal{N}}}. \tag{34c}$$

The form of these equations suggests an interpretation in terms of a spherical coordinate system, where ξ and θ are the azimuthal and polar coordinates and P_{3D} is the radial one. If σ_ξ, σ_θ and $\sigma_{p_{\mathrm{3D}}}$ are the widths (assumed as small) that determine the precision in each of the three coordinates, the element of volume would be $\sigma_{\mathrm{Dir}} = P_{\mathrm{3D}}^2 \sin\theta\, \sigma_{P_{\mathrm{3D}}}\, \sigma_\theta\, \sigma_\xi$, which can be found to simplify to

$$\sigma_{\mathrm{Dir}} \approx \mathcal{N}^{-3/2}. \tag{35}$$

In real measurements, in addition to the measured PSFs there is typically a fairly uniform background. This background also contributes to the noise (assumed here to be Poissonian), and

hence must be considered in the calculations for the CR lower bounds. To account for its effect, we add this background to the intensity distribution in Eq. (24), so that it enters both in the numerator and denominator of the probability density in Eq. (26). This modification causes a few changes in the estimate of the Fisher information matrix in Eq. (28): first, the number of photons used must be that of the signal plus background, but some of the prefactors that are extracted from the sum over pixels due to the change of normalization of $\mathcal{P}$ amount to the fraction of all photons that are due only to the signal. The combination of these two changes then can be combined into a new $\mathcal{N}$ that can interpreted as the number of signal photons. The second effect is that the background must be added to $\mathcal{I}_0$ in the denominator inside the large angular brackets of the last factor in the right-hand side of Eq. (28). Since the average of $\mathcal{I}_0$ over the region occupied by the PSFs is approximately half its peak value, and since the SBR is defined as the ratio of the peak of the PSF to the background, then this change in normalization can be approximated by a factor of $(1 + 2\,\mathrm{SBR}^{-1})^{-1}$. The CR lower bounds then must be multiplied by a factor of $\sqrt{1 + 2\,\mathrm{SBR}^{-1}}$. This approximation is validated by rigorous calculations of the six CR lower bounds for both the directional and spatial parameters, as shown in Supplementary Fig. 3(a). The expression for the global directional lower bound σ_{Dir} then acquires a factor of $(1 + 2\,\mathrm{SBR})^{3/2}$, as described in the main body. Supplementary Fig. 3(b) shows a histogram of $\widetilde{\mathcal{N}}^{3/2}\,\sigma_{\mathrm{Dir}}$ (where $\widetilde{\mathcal{N}} = \mathcal{N}/(1+2\mathrm{SBR}^{-1})$) calculated rigorously for 10000 randomly generated cases with $\mathrm{SBR}^{-1} \in [0, 3]$, and for statistically uniform sampling of the directional parameter space $(P_{3\mathrm{D}}, \xi, \theta)$. We can see that this histogram is indeed peaked near unity.

Let us now consider the Fisher information matrix for both the spatial and directional pa-

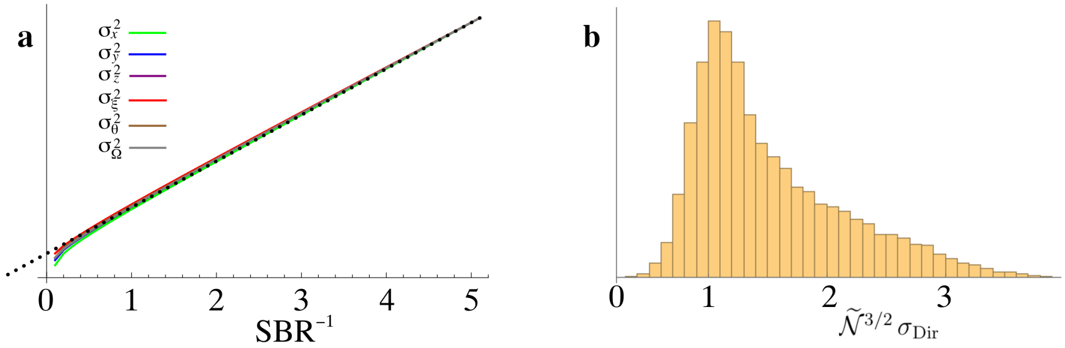

Supplementary Figure 3: Dependence of Cramèr Rao lower bounds on signal-to-background ratio, and global measure of directional/wobbling precision. (a) Squares of the CR lower bounds for the position and direction parameters, as functions of SBR^{-1}, for a non-wobbling fluorophore with $\xi = 0$, $\theta = \pi/2$, and $x = y = z = 0$. All plots were rescaled to show their proportionality to $1 + 2\,\mathrm{SBR}^{-1}$ (dotted line). (b) Histogram of $\widetilde{\mathcal{N}}^{3/2}\,\sigma_{\rm Dir}$ calculated rigorously for 10000 randomly generated cases with $\mathrm{SBR}^{-1} \in [0, 3]$ and statistically uniform sampling of the directional parameter space $(P_{\rm 3D}, \xi, \theta)$.

rameters, $\mathcal{J}^{\rm all}_{\alpha_i\alpha_{i'}}$, for $\alpha_{1,2,3,4,5,6} = x, y, z, \xi, \theta, \Omega$. The coupling between the parameters can be characterized by a normalized version of this matrix, given by

$$C^{\rm all}_{\alpha_i\alpha_{i'}} = \frac{\mathcal{J}^{\rm all}_{\alpha_i\alpha_{i'}}}{\sqrt{\mathcal{J}^{\rm all}_{\alpha_i\alpha_i}\mathcal{J}^{\rm all}_{\alpha_{i'}\alpha_{i'}}}}. \tag{36}$$

The closer this matrix is to the identity, the lower the level of coupling between the parameters. Supplementary Fig. 4 shows both the average and standard deviation (element by element) of this matrix, averaged over all possible directions and over $z \in [-200 \text{ nm}, 200 \text{ nm}]$, for different values of $P_{\rm 3D}$, and both without background and with a SBR of $1/3$. The coupling has negligible systematic bias, so average tends to a diagonal matrix. The standard deviations of the non-diagonal elements do grow when there is more wobble and/or background, but remain, for the chosen parameters, significantly below unity.

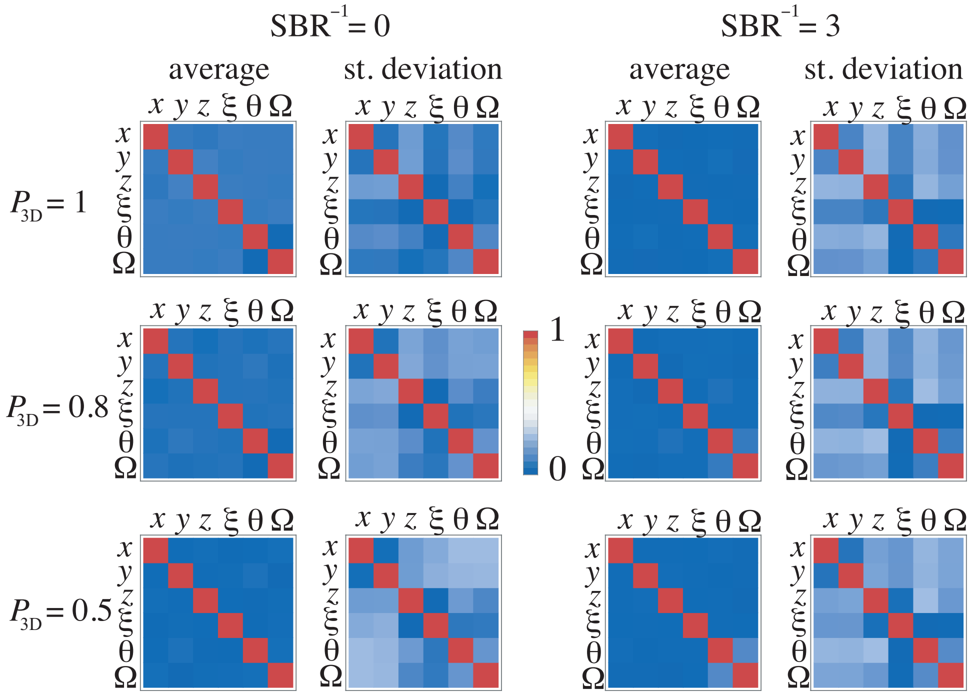

Supplementary Figure 4: Level of coupling in the estimation of different parameters. Averages and standard deviations of the elements of the correlation in Eq. (36) in the absence of background and for a SBR of $1/3$, for $P_{3\mathrm{D}} = 1, 0.8, 0.5$ (namely $\Omega = 0, 0.28\pi, 0.76\pi$).

Finally to show the robustness of CHIDO to aberrations, we repeat the CRB simulations in Fig. 4 of the main manuscript for the case in which the system has one wave of spherical aberrations. The results are shown in Supplementary Fig. 5.

In order to assess the bias (accuracy) and standard deviations (precision) in the estimation of the parameters intrinsic to the method, we simulated numerically PSF pairs with 10000 photons for a range of different parameter values. Given the Poissonian nature of the noise, the optimal retrieval method would be the maximization of the likelihood. However, the method used here, which was easier to implement, was the minimization of the RMS error between the model and the simulated noisy PSFs or, equivalently, the maximization of the normalized correlation between them.

To compare the results with the CR bounds, we considered the same scenarios as in Fig. 4 of the main manuscript, each with 5000 realizations. Supplementary Fig. 6(a-d) shows the standard deviations of the estimations with no background, while (e-h) show the corresponding results for a SBR of $1/3$. Even though the estimation method is not ideal for Poisson noise, the precision obtained agrees well with that predicted from the CR bound analysis, the measured standard deviations being in most cases within a factor of about 3 from these bounds. Note that, for the wobble angle Ω the standard deviations from the parameter retrieval are sometimes smaller that the CR bound. This is because the range for this parameter is finite (and non-periodic) and we are considering cases at an edge of this range. The results are then squeezed against the edge of the allowed interval, giving a distribution that is narrower than what is predicted by the CR analysis.

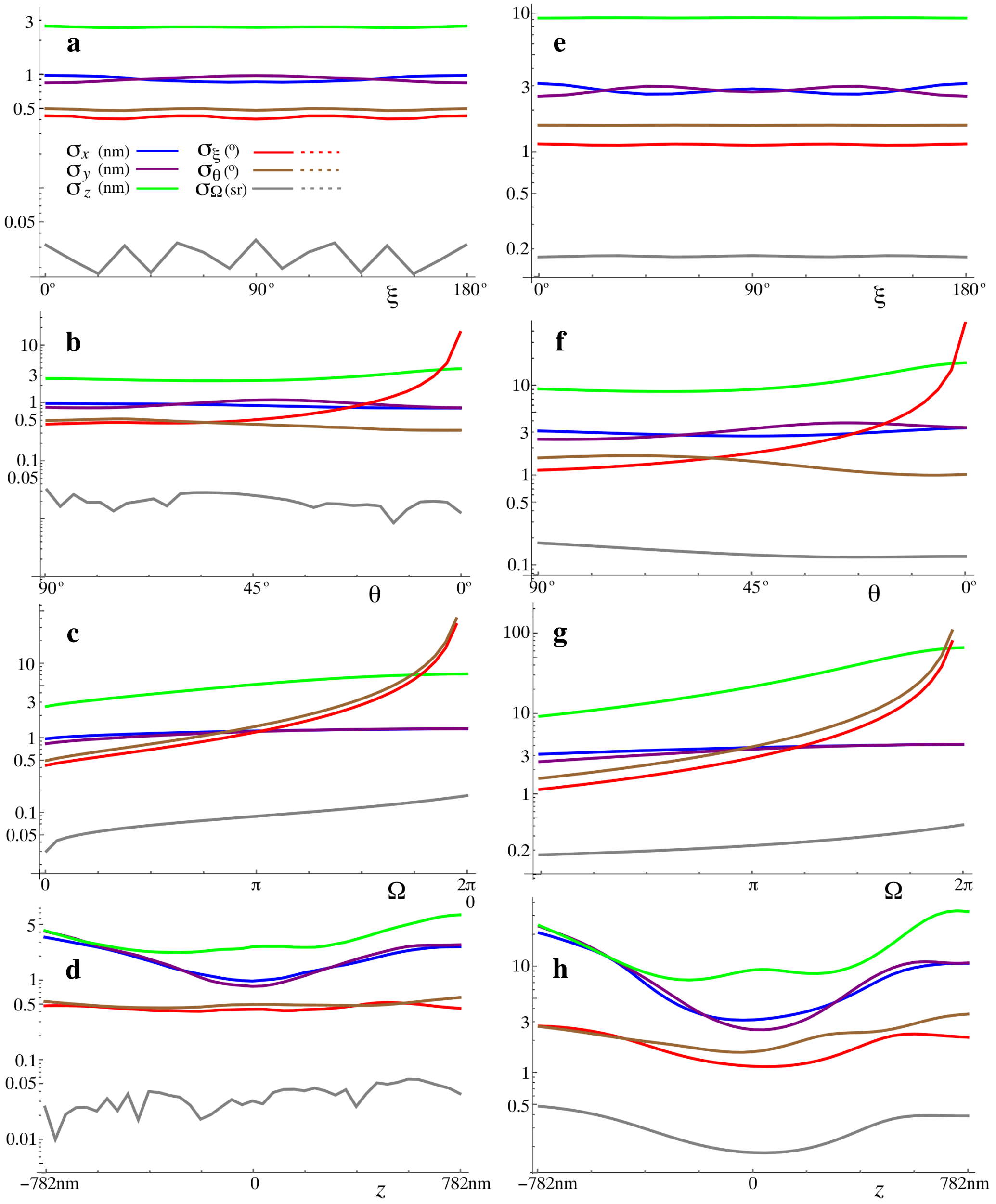

Supplementary Figure 5: Cramér-Rao lower bounds for the six measured parameters when the system presents one wave of spherical aberration. These plots assume 10000 signal photons over the two channels, for (a-d) no background photons, and (e-h) a SBR of 1/3. The parameters are: (a,e) $x = y = z = 0$, $\theta = 90°$, $\Omega = 0$ and varying ξ; (b,f) $x = y = z = 0$, $\xi = 0°$, $\Omega = 0$ and varying θ; (c,g) $x = y = z = 0$, $\xi = 0°$, $\theta = 90°$, and varying Ω; and (d,g) $x = y = 0$, $\xi = 0°$, $\theta = 90°$, $\Omega = 0$ and varying z. The units for each curve are indicated in the legend in (a). The origin in z was shifted by 300 nm, to the plane where the PSFs are the most localized.

As discussed in what follows, proximity to the edge also causes a bias away from it, comparable in size to the standard deviation.

Regarding accuracy, no statistically meaningful biases were found, the average retrieved values being typically of the order of the standard deviation divided by the square root of the number of test cases being averaged. The average deviations from the true value are shown in Supplementary Fig. 7 for the same eight cases as in Supplementary Fig. 6. As mentioned earlier, only for Ω do we see significant biases, but these are caused by the fact that this parameter is defined within a finite interval: for values of this parameter within a few standard deviations from the edges of the interval, there is a small bias away from the edges, of the order of the standard deviation, since the estimation only allows errors to one side of the edges. Note that these figures were generated by using a dense sample of values of the parameter in question, and then averaging cases within small intervals.

Supplementary Note 4: Retrieval of parameters

In this supplementary note we describe a fast approach for the retrieval of the parameters when a basis of PSFs associated with the generalized Stokes parameters and the coefficients of an expansion in z is used. If more accurate results are needed, these parameter estimates can be used as starting points in a more rigorous parameter retrieval routine through the maximization of the likelihood function or the minimization of the rms error. Supplementary Fig. 8(a) shows a theoretically calculated basis for $p = \mathcal{R}$ for a quadratic ($M = 2$) expansion in z. The corresponding

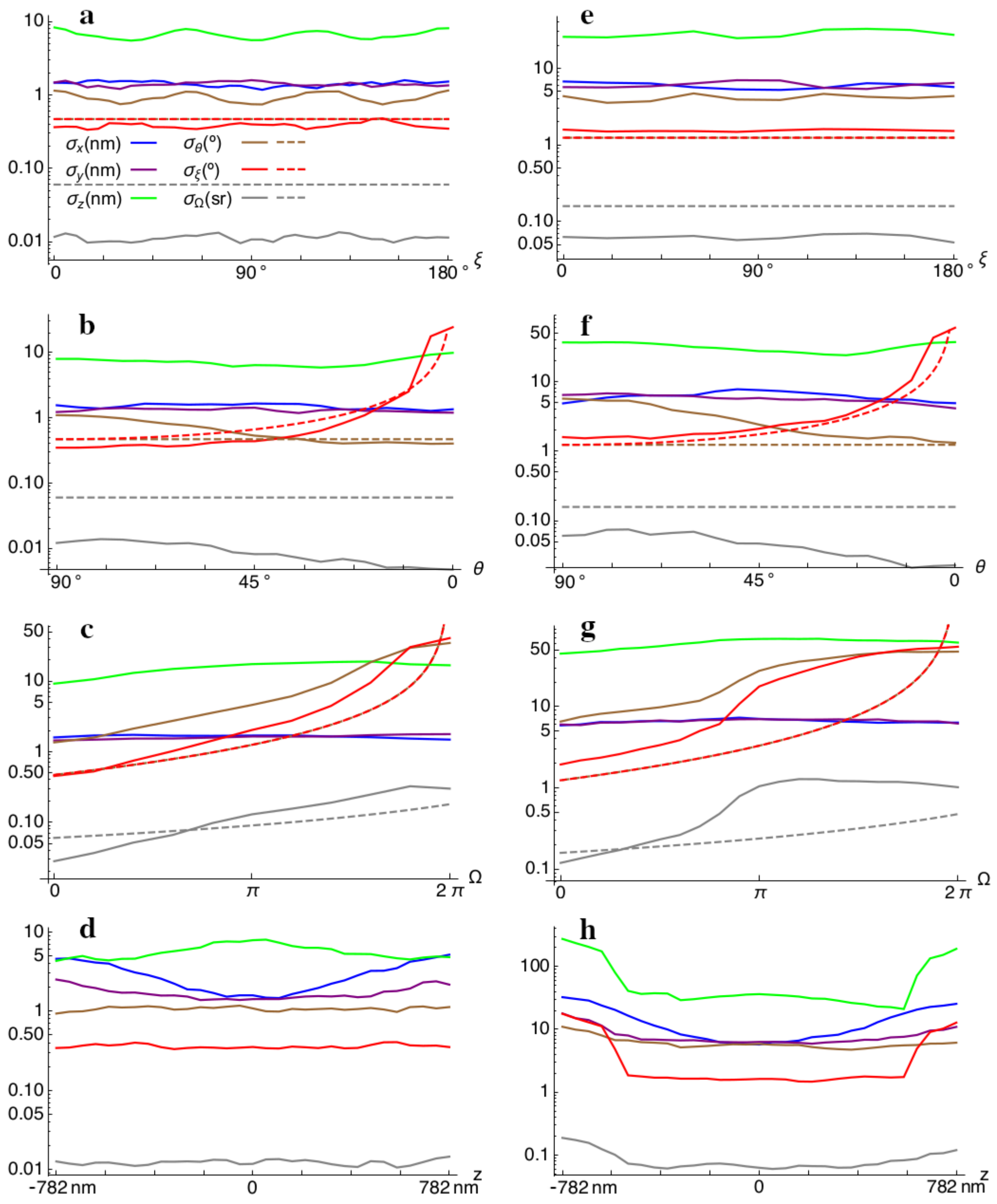

Supplementary Figure 6: Standard deviations for the retrieval of the six measured parameters of numerically-simulated noisy PSFs. These simulations assume 10000 signal photons over both channels, for (a-d) no background photons and (e-h) a SBR of $1/3$. For each scenario 5000 random realizations were simulated. The parameters are: (a,e) $x = y = z = 0$, $\theta = 90°$, $\Omega = 0$ and varying ξ; (b,f) $x = y = z = 0$, $\xi = 0°$, $\Omega = 0$ and varying θ; (c,g) $x = y = z = 0$, $\xi = 0°$, $\theta = 90°$, and varying Ω; and (d,g) $x = y = 0$, $\xi = 0°$, $\theta = 90°$, $\Omega = 0$ and varying z. The units for each curve are indicated in the legends of (a,e). The dashed lines indicate the simple estimates given in Eqs. (34).

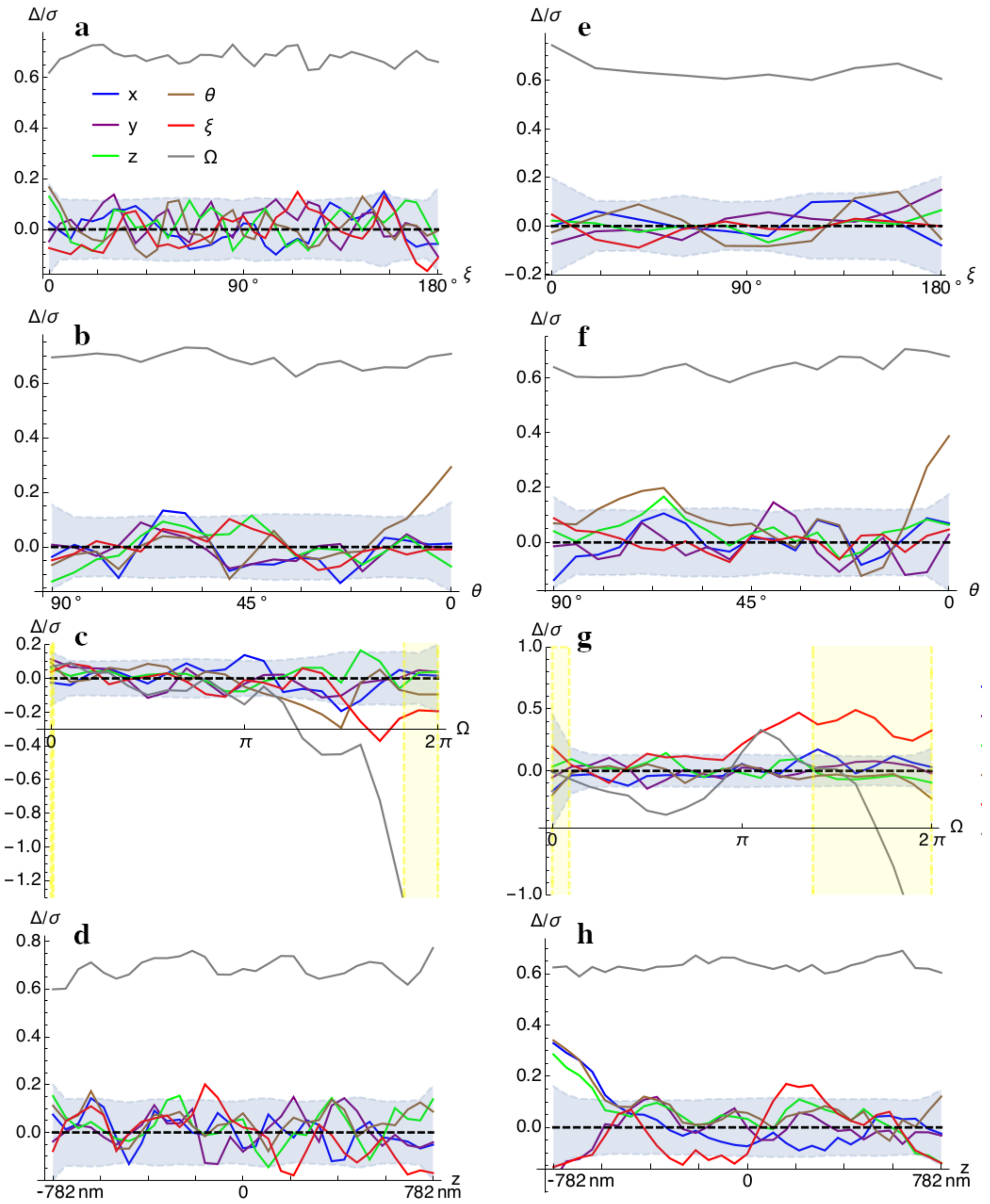

Supplementary Figure 7: Assessment of bias in the estimation of the parameters. Averaged deviation from the true value for each parameter, normalized by the corresponding standard deviation, for each of the cases presented in Supplementary Fig. 6. The dashed thick line indicates zero bias. The blue region corresponds to $\pm 2/\sqrt{n_{\text{samples}}}$, and values that do not deviate far beyond this region can be regarded as statistical fluctuations rather than biases. For (c,g) the yellow regions indicate values of the parameter within $2\sigma_\Omega$ from the edges of the allowed interval for Ω.

PSFs for $p = \mathcal{L}$ are the same except for a sign change in some of its components (encloded in red in the figure). Note that the SEO's orientation was chosen to coincide with the orientation used in the bead measurements, for which the estimated basis, shown in Supplementary Fig. 8(b), was constructed according to a method discussed later.

The determination of transverse position is performed by working in the (discrete) Fourier domain, which also facilitates accounting for the pixelization of the images. The idea is to find the superposition of displaced versions of the PSFs that agrees the most with the measured intensity. This translates into finding the dispacements x, y and the coefficients $S_{n,m}$ for the PSF basis elements $\mathcal{I}_{n,m}^{(p)}$ that minimize the merit function

$$\mu = \sum_{p=\mathrm{r,l}} \left\langle \left(\widehat{\mathcal{T}}_{-x,-y} I^{(p)} - \sum_{n,m} S_{n,m} \mathcal{I}_{n,m}^{(p)} \right)^2 \right\rangle, \tag{37}$$

where $\langle \cdot \rangle$ denotes sum over all pixels, and $\widehat{\mathcal{T}}_{x,y}$ indicates a translation in x and y, which can be implemented in Fourier space as a linear phase and is therefore not constrained to integer multiples of the pixel size. Note that, for convenience, we applied the displacement (with opposite signs) to the measured intensity rather than to the PSF basis functions.

Consider first the solution for the coefficients $S_{n,m}$. These are found by setting to zero the derivative of μ with respect to each of these coefficients, leading to a set of equations of the form

$$\sum_{n',m'} S_{n',m'} a_{n',m',n,m} = b_{n,m}(x, y), \tag{38}$$

where

$$a_{n',m',n,m} = \sum_{p=\mathrm{r,l}} \left\langle \mathcal{I}_{n',m'}^{(p)} \mathcal{I}_{n,m}^{(p)} \right\rangle \tag{39a}$$

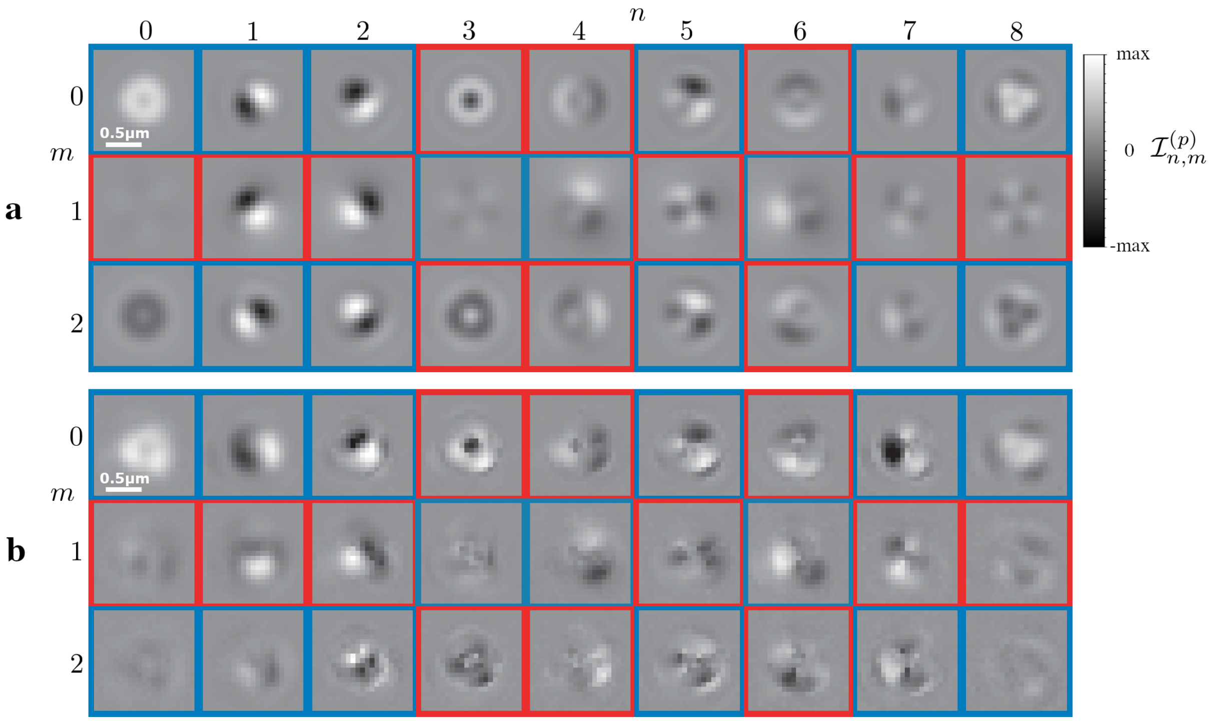

Supplementary Figure 8: Comparison of a theoretical PSF basis and one constructed from a mixture of reference measurements with fluorescent beads and theory. (a) Theoretically calculated PSF components $\mathcal{I}^{(p)}_{n,m}$, with $m = 0$ for PSFs at $z = 0$, for the detector capturing the RHC polarization components ($p = \mathcal{R}$), using $c = \pi$ and assuming $t_\mathrm{s} = t_\mathrm{p} = 1$. For LHC polarization ($p = \mathcal{L}$), the PSF components are the same except that those enclosed by red frames change sign, while the ones enclosed by blue frames remain unchanged. Each row is normalized separately, as the units are different. The SEO is rotated by an angle of $-54.4°$, which fits with the experimental measurements. (b) Corresponding PSF components by using a mixture of experimental and theoretical data.

can be thought of as the elements a $6(M+1)\times 6(M+1)$ matrix $\mathbf{a}$, where M is the maximum value of m, and

$$b_{n,m}(x,y) = \sum_{p=\mathrm{r,l}} \left\langle \mathcal{I}^{(p)}_{n,m} \widehat{\mathcal{T}}_{-x,-y} I^{(p)} \right\rangle$$
$$= \sum_{p=\mathrm{r,l}} \mathcal{F}^{-1}[(\mathcal{F}I^{(p)})^*(\mathcal{F}\mathcal{I}^{(p)}_{n,m})](x,y), \tag{39b}$$

are the correlation of the measured intensity with each of the basis elements. Note that the left-hand side of Eq. (38) can be interpreted as the product of $\mathbf{a}$ and the vector whose components are the coefficients $S_{n,m}$. This equation can be easily solved for these unknown coefficients by finding $\mathbf{a}^{-1}$ and multiplying it by the vector whose elements are $b_{n,m}(x,y)$, namely

$$S_{n,m} = \sum_{n',m'} b_{n',m'}(x,y)\{\mathbf{a}^{-1}\}_{n',m',n,m}. \tag{40}$$

Finally, notice that the substitution of this solution into Eq. (37) gives

$$\mu = \sum_{p=\mathrm{r,l}} \langle [I^{(p)}]^2 \rangle - f(x,y), \tag{41}$$

where the explicitly real and positive function f is defined as

$$f(x,y) = \sum_{n,m,n',m'} \{\mathbf{a}^{-1}\}_{n',m',n,m}\, b^*_{n,m}(x,y)\, b_{n',m'}(x,y). \tag{42}$$

Therefore, the merit function μ is minimized by maximizing the function $f(x,y)$ in x and y. Note that this function is given in terms of the correlations in Eq. (39b), which can be calculated through fast Fourier transforms. The location in x, y of the maxima (corresponding to the transverse position of the emitter) can be determined with accuracy well below a pixel by interpolating via zero padding in the Fourier domain and/or by using a polynomial fit using the values of the pixels surrounding the one with the maximum value.

Finally, notice that the procedure just described allows finding the centroids of multiple emitters in an image (as long as these are well separated), whose positions are given by the local maxima of $f(x, y)$. Once these coordinates are found for each emitter, they can be substituted into Eq. (40) to find the coefficients $S_{n,m}$ and from them the position in z and the orientation and wobbling of the emitter (see next Section). For this final retrieval of the parameters, it is a good idea to use only the section of the images that contains the measured PSF in question. Finally, μ (after appropriate normalization) provides a measure of the quality of the fit, and can therefore be used as a measure of confidence in the results. This measure was used to filter out result where the PSFs overlapped, were too noisy, or were clipped by the edge of the field of view.

As discussed in the main manuscript, fluorescent beads in combination with either a linear polarizer or a radial polarization waveplate (S-waveplate) before the SEO were used to simulate molecule orientations within the xy-plane and in the z direction, respectively. For the first case, the reference PSFs were obtained by choosing a particularly bright and well isolated bead from the second set of measurements. Measurements were taken for different orientations of the linear polarizer over a range of $180°$, at steps of $10°$ (a total of 19 measurements), and at five defocus distances with a spacing of 200 nm. The polarizer was placed not far from the pupil plane, where light is collimated so that the small amount of wedge in the polarizer does not cause changes in defocus as it is rotated. Instead, this wedge did cause a displacement of the PSFs, which moved along a semicircle as the polarizer was rotated by $180°$. Because the initial and final orientations of the polarizer correspond to the same polarization and hence give rise to the same shape of the PSFs, it was easy to determine the length and orientation of the diameter joining the endpoints

of this semicircular path by correlating the initial and final PSFs. The displacement could then be removed computationally (through multiplication by appropriate linear phases in the Fourier domain, so that displacements by fractions of a pixel were possible), leading to a set of PSFs whose origins are consistent. After this recentering, an array of 21×21 pixels containing the PSFs was selected for each. The 40 pixels at the edge of these arrays were used to calculate the background level of the measurements, which was then subtracted. Also, because the number of photons fluctuated from measurement to measurement, each of the 19 arrays was renormalized to make it consistent with the others. From these 19 measurements a fit was performed that predicted the intensity distribution of any orientation in the xy-plane, and from it it was possible to calculate the corresponding PSF elements $\mathcal{I}_1^{(p)}$ and $\mathcal{I}_2^{(p)}$. This procedure was repeated for all five sets of defocus measurements.

Similarly, a sub-basis was generated that emulates the PSFs of fluorophores normal to the plane, by using a bead chosen from one of the samples (set 3) measured with an S-waveplate at the pupil. Again, an array of 21×21 pixels containing the PSFs was selected for each of the five defocus measurements (also spaced by 200 nm), and the background was subtracted by using the values of the edge pixels.

Approximating the dependence in z of the measured PSFs by a simple quadratic polynomial does lead to errors in the estimation. However, these errors are largely systematic and can be corrected by using reference measurements. Supplementary Fig. 9(a) and Supplementary Movie 2 show the averages and standard deviations of the raw estimates of z for the four sets of beads

imaged with an S-waveplate to simulate a molecule normal to the object plane. We can see that the spacing of the estimates is underestimated particularly away from $z = 0$. In this case, remapping the results through a simple cubic expression leads to the corrected estimates shown in Fig. 5(d) of the main text, which are all spaced by approximately 200 nm. Something similar happens for the bead measurements in which a linear polarizer is used to mimic molecules at different in-plane angles ξ. In this case, the distortion caused by the low degree of the polynomial fit also introduces a small amount of coupling between z and ξ, as shown by the raw results in Supplementary Fig. 9(b) as well as Supplementary Movies 3 and 4. Again, because the error is systematic, it can be largely corrected by applying a simple mapping. In this case the mapping was applied to correct only the measurements of set 2, but as can be seen from Fig. 6(b) of the main text, this correction also fixes significantly the estimates of set 1, except for those for the most negative values of z which fell outside the remapped region.

The measurements with beads had the goal of showing the feasibility of height and in-plane orientation measurements. For the fluorophore measurements, on the other hand, the orientations are not known to be purely in-plane or out-of-plane, and in general there is wobbling. Therefore it is no longer possible to use a sub-basis; the complete basis of PSF elements is required.

For the first set of single molecule measurements (at different heights) the strategy we used was to combine the two sub-bases obtained from the bead reference PSFs. This information is not complete, so it had to be supplemented with theoretical calculations for conforming the basis, as explained in what follows. There were several challenges in combining the sub-bases obtained

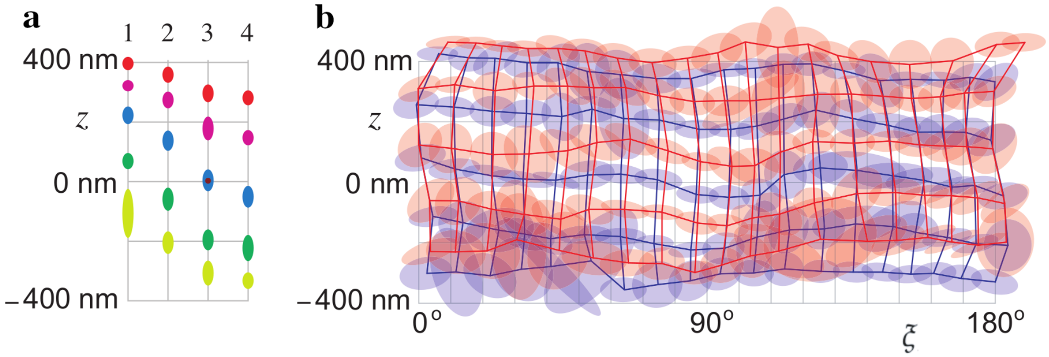

Supplementary Figure 9: Raw estimations of height and in-plane direction for bead measurements. (a) Estimation of z using quadratic approximation (average - center of the ellipse, and standard deviation - height of the ellipse) for the five defocused measurements of the four sets of measurements. All retrieved data are depicted in Supplementary Movie 2. The corresponding data fixed by using a cubic correction is shown in Fig. 5(d) of the main text. (b) The intersection points of the blue and red grids indicate the averages of the raw retrieved heights and orientation angles for each measurement, for sets 1 and 2, respectively, and the ellipses centered at each intersection indicate the corresponding standard deviations. A shift of 16° was applied to the ξ-axis so that the retrieved angles fall in the range $[0°, 180°]$ for ease of interpretation. The full set of data is shown in Supplementary Movie 3 for set 1 and Supplementary Movie 4 for set 2. The corresponding results after the application of a correction to calibrate the results of set 2 (red) is shown in Fig. 6(b) of the main text.

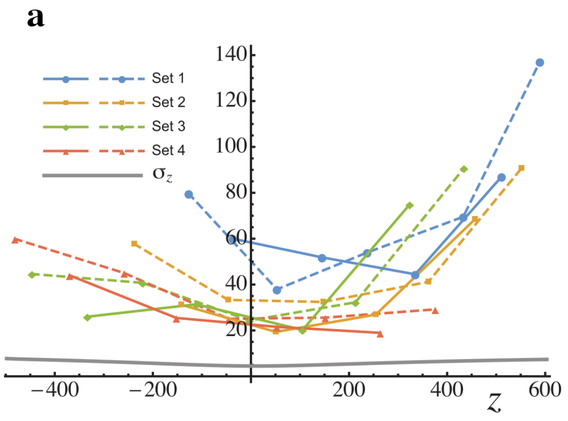

Supplementary Figure 10: Statistical analysis of the estimations of height for bead measurements. (a) For the four sets of bead measurements simulating molecules oriented in the z direction, standard deviations of the z estimate (dashed) and standard deviations of the z increment per bead (solid), where the horizontal axis coordinate corresponds to the average of the z estimates for the corresponding measurements for each set. The gray line corresponds to the CR lower bound calculated using the same model used for the retrieval, for 50000 photons and SBR = 3. (b) For the two sets of bead measurements simulating molecules oriented in several in-plane directions, standard deviations of the z estimate (open dots) and standard deviations of the z increment per bead (solid dots), where the horizontal axis coordinate corresponds to the average of the z estimates for the corresponding measurements for each set. Each dot color represents the measurements for a given height and the different dots with the same color correspond to an image simulated a given molecule direction. The dashed and solid black lines represents the average spreads for the heights and height increments, respectively, while gray line corresponds to the maximum CR lower bound calculated using the same model used for the retrieval, for 50000 photons and SBR = 3.

from the measurements using the S-waveplate and the linear polarizers. First, the two sets of reference measurements are not co-centered in the xy-plane, and in fact there is no guarantee that they are consistent in z either because it is challenging experimentally to know the exact, absolute 3D position of a given bead. Second, the S-waveplate measurements provide distributions that are proportional to those appearing in the definitions of $\mathcal{I}_0^{(p)}$ and $\mathcal{I}_8^{(p)}$ in Eqs. (8a) and (8i), namely $|G_z^{(p)}|^2$. However, the remaining elements of the PSF basis require cross terms between the transverse and axial components, and the rigorous experimental determination of these would require a reference dipole oriented, say, at $45°$ from the z axis, which is not easy to achieve experimentally.

While there are other possible strategies for addressing this issue, the one used here was to combine the measurements with the theoretical model. Some parameters of the theoretical model were adjusted so that the theoretical predictions were as consistent as possible with the reference measurements. One parameter in particular was the orientation of the SEO, which was rotated by an angle of $-54.4°$ with respect to the configuration corresponding to Eq. (1) in the main text. Comparisons between theory and the experimental measurements allowed determining the relation between the x, y, and z coordinates of the two reference sets. Centering with respect to (x, y) was performed by multiplying by the appropriate linear phase factor in the Fourier domain. For each of the two sets, a quadratic fit in z was performed, as described earlier, which allowed defining for each the nominal $z = 0$ value for which the RHC and LHC PSFs are most aligned. The two sets of measurements were then renormalized to be as mutually consistent as possible when compared to the corresponding theoretical calculations. From these results, the magnitudes of $G_i^{(p)}$ for $i = x, y, z$ could be calculated by taking the square root of the corresponding component.

The problem of the missing phases of $G_i^{(p)}$ required for the calculation of $\mathcal{I}_4^{(p)}$ and $\mathcal{I}_6^{(p)}$ was then resolved by using those from the adjusted theoretical models. The resulting PSF basis is shown in Supplementary Fig. 8(b). For comparison, Supplementary Fig. 8(a) shows the theoretically-calculated PSF basis for the estimated SEO orientation.

A different approach was used for the STORM measurements. Given the higher resolution of the camera used for those measurements as well as a higher value of $c = 1.2\pi$, it was better not to use directly the PSFs measured for fluorescent beads because the blurring due to the bead's size was appreciable. Instead, the parameters of the theoretical model were adjusted so that, when blurred by the 3D dimension of the beads, they resembled those measured with beads. This theoretical model was then applied to retrieve the values of the parameters by maximizing their correlation with the measured PSFs. It should also be noted that, because the STORM measurements used a camera with smaller pixels, we used arrays of 29×29 pixels for the references instead of 21×21.

Supplementary References

1. Lieb, M. A., Zavislan, J. M. & Novotny, L. Single-molecule orientations determined by direct emission pattern imaging. *J. Opt. Soc. Am. B* **21**, 1210–1215 (2004).

2. Böhmer, M. & Enderlein, J. Orientation imaging of single molecules by wide-field epifluorescence microscopy. *J. Opt. Soc. Am. B* **20**, 554–559 (2003).

3. Aguet, F., Geissbhler, S., Mrki, I., Lasser, T. & Unser, M. Super-resolution orientation estimation and localization of fluorescent dipoles using 3-D steerable filters. *Optics Express* **17**, 6829–6848 (2009).

4. Backer, A. S. & Moerner, W. E. Determining the rotational mobility of a single molecule from a single image: a numerical study. *Opt. Express* **23**, 4255–4276 (2015).

5. Hieu Thao, N., Soloviev, O. & Verhaegen, M. Phase retrieval based on the vectorial model of point spread function. *J. Opt. Soc. Am. A* **37**, 16–26 (2020).

6. Backer, A. S., Lee, M. Y. & Moerner, W. E. Enhanced DNA imaging using super-resolution microscopy and simultaneous single-molecule orientation measurements. *Optica* **3**, 659–666 (2016).

7. Zhang, O. & Lew, M. D. Fundamental limits on measuring the rotational constraint of single molecules using fluorescence microscopy. *Phys. Rev. Lett.* **122**, 198301 (2019).

8. Zhang, O., Lu, J., Ding, T. & Lew, M. D. Imaging the Three-Dimensional Orientation and Rotational Mobility of Fluorescent Emitters using the Tri-Spot Point Spread Function. *Appl. Phys. Lett.* **113**, 031103 (2018).

9. Brosseau, C. *Fundamentals of Polarized Light* (John Wiley & Sons, Inc., 1998).

10. Samson, J. C. Descriptions of the polarization states of vector processes: applications to ULF magnetic fields. *Geophys. J. R. Astron. Soc.* **34**, 403–419 (1973).

11. Barakat, R. Degree of polarization and the principal idempotents of the coherency matrix. *Opt. Commun.* **23**, 147–150 (1977).

12. Setälä, T., Shevchenko, A., Kaivola, M. & Friberg, A. T. Degree of polarization for optical near fields. *Phys. Rev. E* **66**, 016615 (2002).

13. Alonso, M. A. Geometric descriptions for the polarization for nonparaxial optical fields: a tutorial. Preprint at http://arxiv.org/abs/2008.02720 (2020).
