# Supplementary material for: Birefringent Fourier filtering for single molecule coordinate and height super-resolution imaging with dithering and orientation
Source: Nat Commun. 2020 Oct 20;11:5307. doi: 10.1038/s41467-020-19064-6 (PMC7576605; doi:10.1038/s41467-020-19064-6)
Supplement: Supplementary file 3 — Description of Additional Supplementary Files [file 41467_2020_19064_MOESM3_ESM.pdf]

Description of Additional Supplementary Files:

Supplementary Movie 1: Left: representation of the height, orientation and wobbling of a fluorescing molecule. The figures labeled as RHS and LHS correspond to the theoretically calculated PSFs corresponding to the images of the right- and left-hand-circular polarizations. The movie shows how these PSFs change with the orientation, height and amount of wobbling of the dipole.

Supplementary Movie 2: Measured image pairs for collections of fluorescent beads, where a S-waveplate was placed at the pupil to simulate the radiation pattern of dipoles oriented in the z direction. The movie includes four sets of beads, where for each set five measurements were taken in which the beads were displaced in z at steps of 200 nm. For each frame, superposed on the PSFs are the retrieved *x,y* locations, and the z location is encoded in color. All three quantities are also shown diagrammatically on the 3D figure on the right, which also shows the average measured height, the standard deviation, and the corrected standard deviation with respect to a tilted plane fitted to the 3D positions.

Supplementary Movie 3: Measured image pairs for collections of fluorescent beads in set 1, where a linear polarizer was placed at the pupil to simulate the radiation pattern of dipoles oriented in different directions within the *xy* plane. The directions were varied over a range of 180 degrees, at steps of 10 degrees. For each direction, five measurements were taken in which the beads were displaced in *z* at steps of 200 nm. For each frame, superposed on the PSFs are the retrieved *x,y* locations and in-plane directions, and the *z* location is encoded in color. These quantities are also shown diagrammatically on the chart and 3D figure in the bottom row, which also show the average measured height, its standard deviation, the corrected standard deviation with respect to a tilted plane fitted to the 3D positions, as well as the average and standard deviation of the in-plane angle.

Supplementary Movie 4: Measured image pairs for collections of fluorescent beads in set 2, where a linear polarizer was placed at the pupil to simulate the radiation pattern of dipoles oriented in different directions within the *xy* plane. The directions were varied over a range of 180 degrees, at steps of 10 degrees. For each direction, five measurements were taken in which the beads were displaced in *z* at steps of 200 nm. For each frame, superposed on the PSFs are the retrieved *x,y* locations and in-plane directions, and the *z* location is encoded in color. These quantities are also shown diagrammatically on the chart and 3D figure in the bottom row, which also show the average measured height, its standard deviation, the corrected standard deviation with respect to a tilted plane fitted to the 3D positions, as well as the average and standard deviation of the in-plane angle.

Supplementary Movie 5: First hundred images of a stack of STORM images using CHIDO. The top and bottom sections correspond to the two circular polarization components.
